# Moving singing for lung health online in response to COVID-19: experience from a randomised controlled trial

**DOI:** 10.1136/bmjresp-2020-000737

**Published:** 2020-11-25

**Authors:** Keir EJ Philip, Adam Lewis, Edmund Jeffery, Sara Buttery, Phoene Cave, Daniele Cristiano, Adam Lound, Karen Taylor, William D-C Man, Daisy Fancourt, Michael I Polkey, Nicholas S Hopkinson

**Affiliations:** 1 National Heart and Lung Institute, Imperial College London, London, UK; 2 Respiratory Medicine, Royal Brompton and Harefield NHS Foundation Trust, London, UK; 3 Brunel University London, London, UK; 4 Respiratory Medicine, Imperial College Healthcare NHS Trust, London, UK; 5 Department of Behavioural Science and Health, University College London, London, UK

**Keywords:** complementary medicine, exercise, infection control, viral infection

## Abstract

**Introduction:**

Singing for lung health (SLH) is a popular arts-in-health activity for people with long-term respiratory conditions. Participants report biopsychosocial benefits, however, research on impact is limited. The ‘SLH: Improving Experiences of Lung Disease trial’, a randomised controlled, single (assessor) blind, trial of 12 weeks SLH versus usual care for people with chronic obstructive pulmonary disease (COPD) (n=120) was setup to help to address this. The first group (n=18, nine singing and nine controls) started face-to-face (five sessions) before changing to online delivery (seven sessions) due to COVID-19-related physical distancing measures. As such, the experience of this group is here reported as a pilot study to inform further research in this area.

**Methods:**

We conducted semistructured interviews and thematic analysis regarding barriers, facilitators and key considerations for transitioning from face-to-face to online delivery. Pilot quantitative outcomes include attendance, premeasures and postmeasures of quality of life and disease impact (Short Form 36 Health Survey, COPD Assessment Test score), breathlessness (Medical Research Council breathlessness scale, Dyspnoea-12), depression (Patient Health Questionnaire-9, PHQ-9), anxiety (Generalised Anxiety Disorder-7), balance confidence (Activity specific Balance Confidence, ABC scale) and physical activity (clinical visit PROactive physical activity in COPD tool, combining subjective rating and actigraphy).

**Results:**

Attendance was 69% overall, (90% of the face-to-face sessions, 53% online sessions). Analysis of semistructured interviews identified three themes regarding participation in SLH delivered face to face and online, these where (1) perceived benefits; (2) digital barriers (online) and (3) digital facilitators (online). Findings were summarised into key considerations for optimising transitioning singing groups from face-to-face to online delivery. Pilot quantitative data suggested possible improvements in depression (treatment effect −4.78 PHQ-9 points, p<0.05, MCID 5) and balance confidence (treatment effect +17.21 ABC scale points, p=0.04, MCID 14.2).

**Discussion:**

This study identifies key considerations regarding the adaptation of SLH from face-to-face to online delivery. Pilot data suggest online group singing for people with COPD may deliver benefits related to reducing depression and improved balance confidence.

**Key messages**

**What is the key question?**

- Can Singing for Lung Health (SLH) be delivered online for people with chronic obstructive pulmonary disease? And if so, what are the practical issues and how does the experience compare with face-to-face participation?

**What is the bottom line?**

- SLH appears safe and enjoyable both face to face and online. Barriers for online sessions included digital access and literacy. However, increasing access to those previous unable to physically access sessions is also important. In this pilot, depression and balance confidence appear to show improvements related to participation in an SLH group that transitioned from face-to-face to online delivery.

**Why read on?**

- To our knowledge, this is the first study to assess health impacts of online group singing sessions. Given the physical distancing measures required by the response to COVID-19, there is a need for singing groups and other similar interventions such as pulmonary rehabilitation to be delivered online. This study helps to inform this and future research in the area.

## Introduction

Many people with chronic respiratory disease (CRD) remain highly symptomatic despite optimal pharmacological treatment. Symptoms including exercise limitation, shortness of breath and depression are common.^1–3^ These can be compounded by social isolation and loneliness, which have been shown to be important to respiratory health outcomes.^4^ Group singing is a common practice in most societies globally and has been shown to have health and well-being benefits for people living with long-term health conditions and the wider general public.^5–7^ There is increasing interest in arts-in-health interventions for people with chronic health conditions from patients through to government level.^8^ Singing for lung health (SLH) is a popular group singing programme specifically developed for people with CRD. Small scale trials and qualitative studies suggest SLH has a range of biopsychosocial benefits for participants,^9 10^ however, there is a lack of larger, longer-term, randomised controlled trials (RCTs) regarding the impacts of this intervention.^11 12^ The ‘Singing for Health: Improving Experiences of Lung Disease (SHIELD) trial’ was setup to help to address this gap, planning to randomise 120 individuals with chronic obstructive pulmonary disease (COPD) to participation in 12 weeks of group singing or usual care (UC).

During the current COVID-19 pandemic, physical distancing measures aimed at reducing SARS-CoV-2 transmission have led to profound social adaptations and disruption.^13^ Group activities, particularly involving people with long-term health problems who are especially vulnerable to COVID-19, have in most cases been suspended, including pulmonary rehabilitation (PR) programmes—one of the highest value interventions for people with respiratory disease.^12 14^ Similarly, there are particular concerns that group singing could be a high-risk activity regarding viral transmission, however research is currently limited.^15^ ^15^

This context has driven interest in the implementation, and ongoing development, of online approaches that attempt to reproduce the social, psychological and physical effects of singing, dance and more established interventions such as PR.^16 17^ Such approaches are especially important for people with lung conditions as even prior to the COVID-19 pandemic access to these interventions was inadequate.^18 19^ Furthermore, measures to reduce risk of COVID-19 in this group appear to be causing substantial disruption to care and access to health services, with high levels of anxiety and loneliness being reported.^13 20 21^

The first group of participants in the SHIELD trial, which began in February 2020, initially met face-to-face but the delivery of the intervention changed to an online format which is likely to remain necessary for the foreseeable future. We decided that this transitional group should be reported as a pilot study. This was first because research on the health and well-being impacts of online singing groups is lacking so the results could guide both the further delivery of the SHIELD trial and the design of other studies. Second, this would provide useful information from individuals who had experience of both face-to-face and online activities and could therefore enable a comparison of the two types of intervention experience.

## Methods

### Trial design and oversight

The SHIELD Trial was prospectively registered at ClinicalTrials.gov (NCT04034212). The current analysis was defined as an amendment to the initial trial registration when the delivery of singing moved from face to face to online.

### Participants

**Figure 1:**
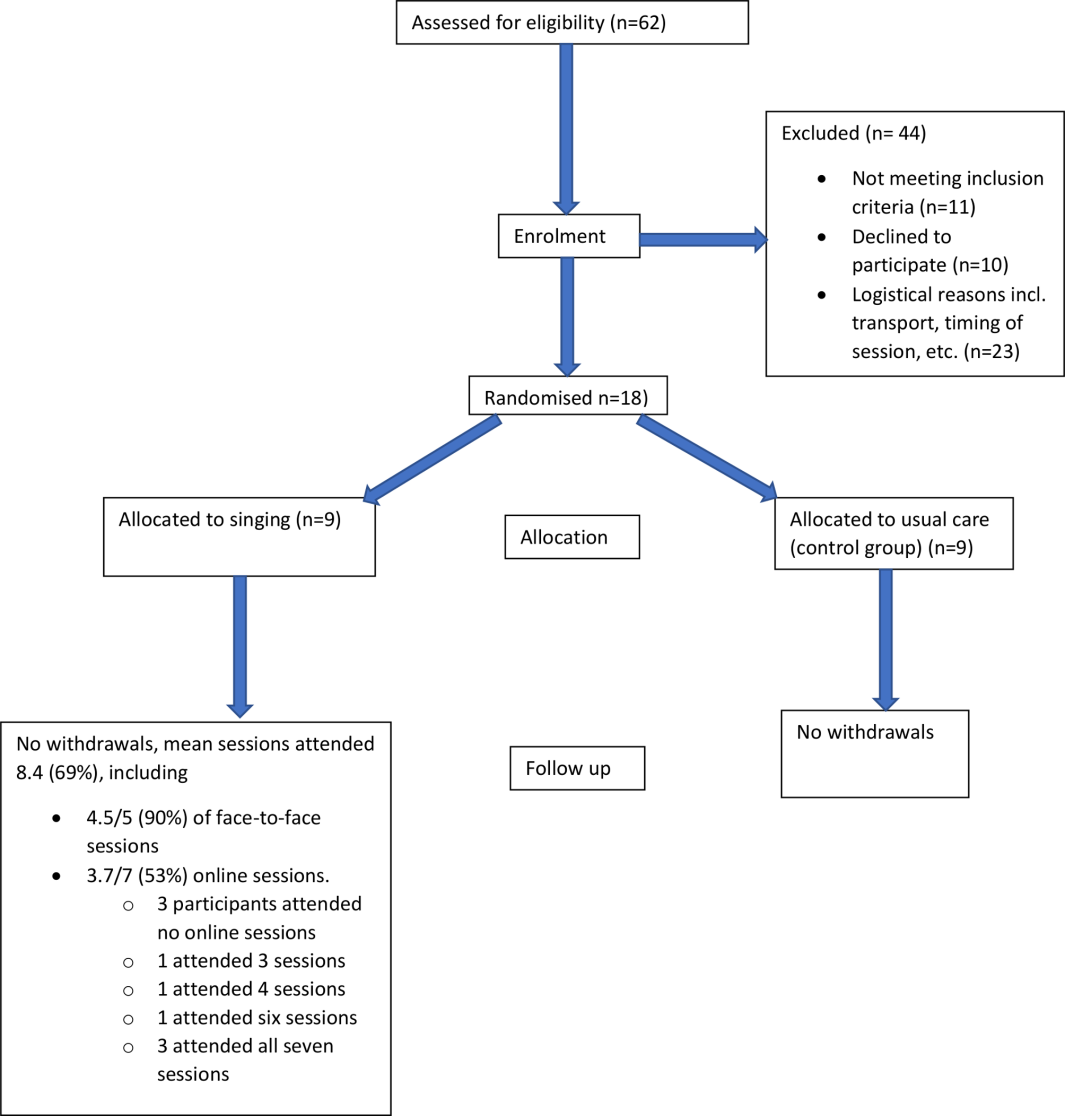
Moving singing for lung health online: experience from a randomised controlled trial: CONSORT diagram. CONSORT, Consolidated Standards of Reporting Trials.

The first group of 18 participants in the SHIELD trial were recruited from a specialist COPD clinic at the Royal Brompton Hospital London and lists of previous research participants who had given consent to be contacted regarding research (figure 1). COPD diagnosis was confirmed by spirometry as per the Global Initiative for Chronic Obstructive Lung Disease (GOLD) guidelines.^22^ Exclusion criteria included pulmonary rehabilitation within the preceding 4 months. The effects of pulmonary rehabilitation tend to wane over time,^23^ and this interval was selected to give a reasonable chance of avoiding the most immediate impact of having done PR on study measures. We did not include people who would have been due to start a PR course within the study period to ensure that there were no delays to UC. Further exclusion criteria included an inability to take part in singing sessions due to comorbidity (eg, life limiting illness, cognitive impairment) and previous participation in SLH classes. Given the requirement for the original protocol of weekly in-person attendance, from the list of potential participants, people living within a 1 hour journey of the hospital (estimated using google maps) were contacted first. All participants provided written informed consent after reading the participant information sheet (PIS) and being given the opportunity to ask questions. Transport costs related to the assessment visit were reimbursed, but no payments were made for participation.

### Patient and public involvement

The Royal Brompton Clinical Research Facilities patient expert and lay person research panel, and the Royal Brompton ‘Breathe Easy’ Group, reviewed the study proposal, provided thoughts and suggested improvements which improved the study design and materials prior to Research Ethics Committee review. In particular, the choice of a quality of life primary outcome measure was well received. The Participant Information Sheet (PIS) was clarified including reducing use of specialist terms and specifying which study-related costs would be reimbursed. Overall, the study was very well received by these patient and public representatives who saw clear value in the patient centred focus of the study.

### Interventions

The intervention arm (SLH) received 12, once weekly, hour long, SLH sessions. The study control arm received UC (no specific additional intervention above those which the person usually engages with). The specific content and structure of the singing sessions has been described elsewhere,^10^ but briefly, each session consisted of a physical warm up, breathing exercises, vocal warms ups, songs and a cool down. The sessions were led by Edmund Jeffery, a professional singing teacher with 4 years experience leading SLH groups. The SLH participants also received a CD of singing exercises and were encouraged to practice between sessions, daily if possible.

The first five, weekly sessions were delivered face to face as originally planned at the Royal Brompton Hospital London, UK, and started on the 10 February 2020. These were halted due to the developing COVID-19 situation in the UK for 2 weeks to develop the online delivery of sessions. This process included discussion within the SLH provider network regarding suitable content, potential barriers and facilitators, and trial sessions with experienced leaders to help address unidentified challenges. The online SLH format which was developed was then used to deliver the final seven sessions via a video conferencing application (Zoom Video Communications, ‘Zoom’).

### Baseline assessment, randomisation and blinding

Assessments took place at the Royal Brompton Hospital (London, UK). Following a screening visit, written informed consent was taken, followed by a structured clinical history, and confirmation of COPD diagnosis by spirometry, and baseline assessment of outcome measures. Participants were randomised (1:1) using computer-generated block randomisation lists (Sealed Envelope) block size 4, stratified by Medical Research Council (MRC) breathlessness grade and previous participation in pulmonary rehabilitation. The outcome assessors were blind to intervention allocation. Blinding of participants was not possible, and they were informed of their allocation by the unblinded researcher responsible for randomisation (SB). Unblinding of assessors took place only after all outcome measure data had been recorded.

### Outcome measures and assessment

Primary outcome measures were change in the Short Form 36 Health Survey (SF-36) Physical and Mental Component Scores using the oblique scoring method,^24^ with subscales provided to aid interpretation. Secondary outcomes included change in SF-36 subscales, balance confidence (Activity-specific Balance Confidence, ABC scale), anxiety (Generalised Anxiety Disorder-7, GAD-7), depression (Patient Health Questionnaire-9, PHQ-9), COPD assessment test (CAT) score and breathlessness (MRC dyspnoea score and Dyspnoea-12). Patient experience of physical activity was assessed using the clinical visit PROactive physical activity in COPD tool (cPPAC). The cPPAC combines questionnaire and 7 days of actigraphy measures to produce two domains, amount and difficulty.^25 26^ Actigraphy data were collected using the Dynaport MoveMonitor (McRoberts BV, The Hague, The Netherlands) which is validated for use as part of the cPPAC tool. Participants were requested to wear the device on a belt around the lumbar region of their back, for 7 days, taking it off for showering/bathing. Full guidance on these devices is available from the manufactures at https://www.mcroberts.nl/.

The original SHIELD protocol included secondary outcome measures of physical capacity and performance testing using the 6 minute walk test (6MWT) and the short physical performance battery (SPPB), respectively. However, these were only conducted at baseline as repeating these measures was not possible due to COVID-19-related physical distancing measures.

Outcomes measures were repeated after 14 weeks (12 weeks intervention plus 2-week pause for adaptation of delivery after week 5 of intervention). Baseline assessments were completed within 4 weeks prior to the intervention arms first singing session. Follow-up assessments were completed within 4 weeks of study completion by participants at home, with activity monitors and questionnaires returned by post. Any missing data (unanswered questions) were addressed by telephoning the participant. Adverse events were not formally assessed as an outcome, but participants were advised to inform the session leaders if they become unwell or had any concerns.

### Qualitative data

Semistructured qualitative feedback interviews were conducted on the phone (by KEJP who has training and experience in qualitative research techniques) with the SLH participants following unblinding of researchers. Interviews focused on overall experience of intervention, positives, negatives and barriers and facilitators to participation (as covered with an approved amendment to the Research Ethics Committee application). Notes were made during the call and a template response form was completed immediately after each call to record participant responses and interviewer reflections. Audio recordings of the interviews were not made. Primarily deductive thematic analysis was used^27^ in which initial codes were developed around barriers, facilitators and perceived impacts of participation, using a combination of biopsychosocial^28^ and randomised pilot study conceptual frameworks.^29^ Comparison of experiences of face-to-face and online groups was also sought.

### Statistical analysis

The power calculation for the original SHIELD trial required 120 participants to show a clinically important difference in the primary outcome (SF-36). Given the circumstances, no power calculation was performed for this revised pilot study. Differences in outcomes between study arms were evaluated using one-tailed t-tests for superiority of SLH over UC or the Wilcoxon rank-sum (WRS) test where data were not normally distributed. Analyses were carried out using Stata V.14 (StataCorp) on an intention-to-treat basis.

Data shown are mean (SD), or if appropriate, median (IQR)/number (%) as indicated. P values are for t tests for between groups differences, or WRS as marked, if appropriate. Body mass index; 6 min walk distance, 6MWT distance; SPPB; prestudy perception of SLH impact, participants asked to rate on scale of 0–10 expected impact of singing on improving health with 0 is no impact, and 10 very large improvement in health; SF-16; PHQ-9; GAD-7 questionnaire; ABC score, Activity specific Balance Confidence scale; MRC; CAT; PROactive, Patient Reported Outcome measure for physical activity in COPD.

## Results

The intervention and control groups were well-matched at baseline (table 1). No serious adverse events were reported by study participants, though these were not sought systematically. No participants withdrew from the study. However, difficulties with attendance at the online sessions is described below. Use of the CD for practice between sessions was not formally quantified, however, participants reported very variable use, from not at all to daily. Summaries are provided regarding considerations (table 2), and practical issues (table 3), relating to transitioning from online versus face-to-face delivery of SLH, based on participant and facilitator experiences.

**Table 1:** Baseline characteristics

	Singing for lung health (SLH) (n=9)	Usual care (n=9)	P value
Age	72.1 (9.65)	69.89 (9.36)	0.63
Female	3 (33%)	6 (66%)	0.18
BMI	23.30 (7.20)	25.52 (6.41)	0.50
FEV_1_ % predicted	56.39 (35.57)	35.01 (17.84)	0.13
Oxygen therapy	1 (11%)	2 (22%)	0.60
Pack years smoked	36.23 (19.95)	41.89 (31.26)	0.66
Falls in last year	1 (11%)	1 (11%)	1.00
Baseline 6MWD	418 (136.23)	366 (82.30)	0.34
SPPB total	9.56 (2.19)	9.11 (2.32)	0.68
Prestudy expectation of SLH health impact (0 ‘no impact’ to 10 ‘large impact’)	4.56 (1.33)	4.56 (2.24)	1.00
SF-36 Physical Component Score	39.11 (8.39)	34.91 (8.26)	0.30
SF-36 Mental Component Score	45.76 (4.60)	41.52 (35.16)	0.20
SF-36 Physical function	43.89 (26.43)	36.67 (27.95)	0.58
SF-36 Role limitation, physical	50.00 (41.46)	25.00 (39.53)	0.21
SF-36 Role limitation, emotional	85.19 (24.22)	62.97 (35.14)	0.14
SF-36 energy	40.56 (17.40)	42.23 (18.05)	0.84
SF-36 emotional well being	74.67 (11.66)	66.23 (14.44)	0.19
SF-36 social functioning	79.17 (20.73)	61.11 (34.60)	0.20
SF-36 pain	65.56 (21.71)	61.39 (29.77)	0.74
SF36 general health	32.22 (13.94)	31.11 (14.95)	0.87
SF-36 health change	47.22 (19.54)	52.78 (23.20)	0.59
Depression (PHQ-9)	5.00 median (IQR 3.00–8.00)	6.00 median (IQR 5.00–14.00)	0.28 (WRS)
Anxiety (GAD-7)	2.00 median (IQR 1.00–2.00)	5.00 median (IQR 0.00–11.00)	0.23 (WRS)
ABC scale score	78.75 median (IQR 50.63–90.63)	76.25 median (IQR 64.38–91.25)	0.72 (WRS)
Dyspnoea 12	11.00 median (IQR 7.00–15.00)	13.00 median (IQR 8.00–20.00)	0.27 (WRS)
MRC dyspnoea score	2.89 (0.93)	3.00 (1.32)	0.84
CAT score	18.11 (7.10)	21.00 (7.57)	0.42
PROactive difficulty*	68.56 (13.28)	63.11 (14.60)	0.42
PROactive amount†	53.67 (14.14)	46.67 (18.21)	0.38
PROactive total‡	61.11 (12.06)	54.89 (13.86)	0.32
Daily step count	2717 median (IQR 1870–4871)	2566 median (IQR 1213–3119)	0.40 (WRS)

Data shown are mean (SD), or if appropriate, median (IQR)/number (%) as indicated. P values are for ttests for between groups differences, or Wilcoxon rank-sum (WRS) as marked, if appropriate.

*Scale of 0 (high level difficulty) to 100 (low level of difficulty).

†Scale of 0 (low amount) to 100 high amount.

‡Total score calculated as mean of PROactive difficulty and PROactive amount (0–100).

ABC, Activity specific Balance Confidence; BMI, body mass index; CAT, COPD Assessment Test; FEV1, forced expiratory volume in 1 s; GAD-7, Generalised Anxiety Disorder-7; MRC, Medical Research Council; 6MWD, 6 min walk distance; PHQ-9, Patient Health Questionnaire-9; PROactive, Patient Reported Outcome measure forphysical activity in COPD; SF-36, Short Form 36 Health Survey; SPPB, short physical performance battery; WRS, Wilcoxon rank-sum.

**Table 2:** Considerations for online versus face-to-face delivery of singing for lung health (SLH) based on participant and facilitator experience

	Face to face	Online
Access: Physical	More challenging Geographically local SLH sessions Transport requirement Financial and time costs Availability and accessibility in context of lung condition and symptoms.	Private physical space still required for participant.
Access: Online	Limited or no requirement Can be used for session organisation.	Computer/device and internet access required. Overcomes multiple face-to-face physical barriers.
Digital literacy	Not required	Required With support can be minimal Could help build skills/confidence facilitating access to other online services.
Infection/health risks	Potential risk of cross infection from participants or infection during transport to/from session.	Not supervised in person Contact details of next-of-kin advisable for potential issues.
Social experience	Very effective and important to participants.	Good (perhaps less than face to face) Building rapport more challenging, especially if have never met in person (new groups/individuals) Specific content to address advised.
Personal experience	Presence of peers can be both supportive and slightly intimidating depending on individual and group dynamic.	The required technological skills can induce mild anxiety if participant not confident/experienced.
Physical engagement	Important aspect of session Journey to/from session also valuable physical activity for some.	More challenging than face to face Requires specific consideration and promotion.
Facilitator experience	Easier to gauge participant emotions and group dynamic.	More challenging to assess if appropriate techniques are being used by participants.

**Table 3:** Practical issues transferring face to face to online based on participant and facilitators experience

Informing participants	People are generally understanding of current requirement for physically distanced sessions.
Joining online	Participants may need support with digital access. Friends and family a good source of support. Dedicated ‘set up sessions’ could help in which session leader talks the participant through setting up, 1 to 1, separately and in advance of the SLH session.
Physical space	Clean, tidy and free from trip hazards.
Sound	Speakers advisable for better volume and sound quality
Feedback	Integration of formal feedback vital to facilitate responsive and participant appropriate sessions. More immediate feedback required from session leader as participants are not able to hear each other sing.
Maintaining/building relationships	Introductions. General catch up/social. Rapport building activities.
Session content	Cannon and multi-harmonies (live)—difficult. Prerecorded content to facilitate multipart songs possible, but complicated. Focusing on what works best in each method of delivery more important than trying to exactly emulate face-to-face sessions online. Discussion between singing leaders using similar participant groups and swapping ‘best practice’ from experience works well.
Keep up to date	The evidence base is in this area is evolving, so ongoing review of relevant research and guidelines is important, including related activities such as pulmonary rehabilitation.^38^
Safety	Ensure contact details are correct. Asking participants to provide next of kin details can be useful. Ensure participants take breaks or stop if feeling unwell in any way. Clear space, free from trip hazards important for participants.
Ethical Issues	Participants should consent to any use of their personal data. Closed/password-protected online sessions important to prevent uninvited interruptions.

SLH, singing for lung health.

### Attendance

For the SLH arm, mean number of sessions attended was 8.4 (69%) of the 12 total sessions. This included a mean of 4.5 (90%) of the five face-to-face sessions, and 3.7 (53%) of 7 online sessions. Three of the participants did not attend the online sessions at all, three attended all seven sessions, with the remaining three participants attending three, four and six sessions. See consort diagram (figure 1).

### Effect on intervention

Mean difference from baseline was compared between the control and SLH groups in table 4 using t-tests, or WRS tests as appropriate. These outcomes are presented for information, but cannot, of course, be used to make any confident inference about the effectiveness or otherwise of the intervention given the limited sample size and lack of statistical power. Comparing singers with non-singers there were statistically significant improvements in the PHQ-9 depression score (treatment effect −4.78 points, p=0.049, Minimum Clinically Important Difference (MCID) 5) and ABC scale for balance confidence (treatment effect +17.21 points, p=0.038, MCID 14.2).

**Table 4:** Comparison of change in outcome measures between study arms

	Singing for lung health (SLH) (n=9)	Usual care (UC) (n=9)	Treatment effect (95% CI)	P value
SF-36 Physical Component Score	−1.66	−0.389	−1.27 (−7.13 to +4.60)	0.67
SF-36 Mental Component Score	−0.367	−4.30	+3.93 (−3.85 to +11.72)	0.15
SF-36 Physical function	−1.11	2.77	−3.89 (−14.02 to +6.24)	0.79
SF-36 Role limitation, physical	−16.67	−2.78	−13.89 (−42.44 to +14.66)	0.84
SF-36 Role limitation, emotional	−7.41	−33.33	+25.92 (−14.51 to +66.35)	0.10
SF-36 energy	6.11	−1.11	+7.22 (−9.59 to +24.03)	0.19
SF-36 emotional well-being	2.22	−6.22	+8.44 (−7.62 to +24.51)	0.14
SF-36 social functioning	−6.95	−15.28	+8.33 (−18.85 to +35.52)	0.26
SF-36 pain	0.28	5.78	−5.50 (+29.57 to +18.57)	0.68
SF-36 general health	0	1.67	−1.67 (−12.98 to +9.65)	0.62
SF-36 health change over last year	5.56	−13.89	+19.44 (−0.09 to +38.97)	**0.026**
Depression (PHQ-9)	−1.44	+3.33	−4.78 (−10.53 to +0.98)	**<0.05**
Anxiety (Median (IQR))	0.00 (0.00 to 1.00)	2.00 (0.00 to 3.00)	2.00	0.24
ABC scale score	6.03	−11.18	+17.21 (−2.07 to +36.49)	**0.04**
Dyspnoea 12	−0.445	0.445	−0.89 (−7.69 to +5.91)	0.39
MRC dyspnoea score	0.222	0.111	+0.11 (−0.61 to +0.84)	0.63
CAT score	−1.44	2.22	−3.67 (−9.42 to +2.08)	0.10
PROactive Difficulty*	0.889	−1.12	+2.00 (−9.78 to +13.79)	0.362
PROactive amount†	−20.22	−6.89	−13.33 (−30.07 to +3.41)	0.95
PROactive total‡	−9.67	−4.00	−5.67 (−13.68 to +2.35)	0.92
Daily step count (median IQR))	−531 (–823–-65)	−372 (−1123–150)	−159	0.76

P values are one-tailed independent sample t-tests of mean change preintervention–postintervention, for superiority of SLH over UC, or Wilcoxon rank-sum test if non-parametric test required, with median (IQR) shown.

*Change in the scale of 0 (high level difficulty) to 100 (low level of difficulty);.

†Change in the scale of 0 (low amount) to 100 high amount.

‡Change in the scale of the total score calculated as mean of PROactive difficulty and PROactive amount (0–100). MCID for the PHQ-9 is 5 points^39^; MCID for ABC score is 14.2 points.^40^

ABC, Activity specific Balance Confidence; CAT, COPD Assessment Test; MRC, Medical Research Council; PHQ-9, Patient Health Questionnaire-9; SF-36, Short Form 36 Health Survey.

### Participant experience

Eight of the nine SLH participants were interviewed by phone, one was not available. Deductive analysis identified three key themes regarding the SLH participants’ experience:

1. Perceived benefits.
2. Digital barriers.
3. Digital facilitators.

#### Perceived benefits

All SLH participants reported greatly enjoying participation while session delivery was face to face. The online sessions were enjoyed by the majority of participants, though all stated an overall preference for face-to-face sessions. This preference related primarily to the social environment and interpersonal interactions. Participants spoke fondly of their interactions with each other when face to face but highlighted that this was more difficult to achieve online. Participants emphasised that these social aspects were particularly important due to the social distancing measures that had been put in place due to COVID-19. It was frequently remarked on that the sessions provided an important connection to the ‘outside world’. Participants reported it ‘helps your breathing’ and that ‘Certainly my breathing is better now than before’ especially due to breathing control exercises and techniques. Improvements in mood and enjoyment of the social aspects were frequently reported. There was also a perception that the singing had contributed to other types of physical activity ‘the singing has contributed to my exercise levels’. Such benefits were seen as important due to their relevance to symptoms of their lung condition.

#### Digital barriers

Barriers relating to online delivery mostly related to technical difficulties. The majority experienced some form of technical difficulties; only those who reported regularly (weekly or more), and independently, using online conferencing tools had no issues. Of those that did not attend at all online, one did not have a computer and one did not have a functioning internet connection. The third participant who did not participate online chose not to as they felt making noise would not be considerate to their neighbours, given the limited sound insulation, and ongoing ‘lockdown’ measures for all the residents of his building. Some participants were able to ask friends or family members to help with overcoming technological challenges, which worked well. However due to the ‘lock-down’ and social shielding, this was not possible for all the participants. Some of the technological difficulties experienced by participants appeared potentially addressable with appropriate support. However, some participants seemed unconfident in trying to address them. A lack of confidence trouble-shooting technological challenges is likely to have consequences beyond these sessions by limiting access to digital health and social resources more generally. As such, supporting digital access in this context could be a useful way to build digital literacy and confidence.

Online delivery was also felt to be less personal, as interaction between participants was more challenging *‘*meeting at the Brompton was much more engaging. It’s so nice to sing together as a group’. One participant stated that ‘Physically demanding things are better done in a group*’*, with group motivation more palpable in person than online. The extent to which this preference for face-to-face interaction was modulated by the COVID-19-related social distancing measures and fear for personal safety^13^ is difficult to tell. It is possible that the relative, or even complete, absence of face-to-face interaction in any aspects of the participants lives during the ‘lockdown’ heightened the value that they attributed to the face-to-face singing sessions they attended at the beginning of the study. Even though face to face was preferred, online delivery was still seen as being extremely valuable ‘Even online, it’s an up-lifting thing to do for mental health. We spent quite a lot of time laughing. Singing as a group is special.’ Other aspects of the sessions were also noted to be lost when adapting the sessions to online. For example ‘singing in canon (a compositional technique in music)*’*, which was thoroughly enjoyed in person, were not technically possible during sessions delivered online.

It was felt that by having started the sessions face to face a degree of rapport had been built between participants, and with the singing leader, that helped with the transition online. It was suggested that it might be more difficult to establish rapport between group members if groups commenced online with no prior meeting in person.

#### Digital facilitators

On certain points, online delivery was perceived as having benefits over in person sessions. One participant highlighted that ease of attendance enabled them to attend when they were not feeling 100% well and would not have attended in person had they needed to physically transport themselves to the singing session. Another participant felt that by being online they were less self-conscious singing with the other participants as they would not be able to hear the participant’s voice. Multiple participants highlighted that online delivery overcame many of the barriers related to physically accessing face-to-face participation including geographical location of sessions, using transport with high symptoms burden, and current infection risks.

## Discussion

To our knowledge, this is the first study to assess the practical delivery of an online group singing intervention for people with respiratory disease intended to improve health and well-being. This transition from face-to-face to online delivery was forced on us by the COVID-19 pandemic, but provides useful information about how this can be done and allowed us to gain insights from people who had experienced both formats of delivery.

Key findings include that SLH delivered online was viewed as enjoyable and holistically beneficial to health, though face to face was generally preferred. Importantly, the perceived benefits were directly related to moderating their lung condition symptoms. The psychosocial impacts were highly valued by participants, but more difficult to achieve in the online format. Technological difficulties prevented some people from participation in online sessions, which were also felt to be less personal as social interaction was more challenging. The pilot data suggest that group singing for people with COPD, adapted to online delivery, may still deliver benefits related to reducing depression and improved balance confidence.

These findings are broadly supportive of other related studies. However, these findings should be interpreted within the context of COVID-19-related physical distancing and ‘shielding’ measures. In a small (n=28) RCT of a 6-week course of twice weekly face-to-face SLH, Lord *et al*^30^ found psychological improvements, though these related to anxiety rather than depression, and qualitative research has reported similar findings to the current study in relation to perceived impact on health and wellbeing.^31^ Qualitative research from a dance group for people with CRD and breathlessness also identified perceived holistic benefits with an emphasis on psychosocial impacts (KEJP Dance qual). Previous studies on SLH for people with COPD have suggested improvements in quality of life,^30 32 33^ which was not seen here, though it is not clear if this was due to the small sample size. Furthermore, our findings echo those of a study comparing the experiences of participants in a virtual choir with those in a live choir, which found the two types of experience provide very similar emotional benefits, though differences in how ‘present’ participants felt.^34^

The qualitative data identified specific barriers and facilitators related to the different formats of delivery, which helps to explain the attendance data. The consort diagram highlights that many (n=23) potential participants declined due to issues with physically accessing the face-to-face sessions, mainly related to viewing public transport as too challenging given their health condition. However, online delivery demonstrated good potential to overcome physical distance as a barrier to access. As highlighted in the interview feedback, during the online section of the study, there were days when individuals participated but felt they would not have felt well enough to come in person if the sessions were still being held at the hospital. Regarding the online sessions, difficulties with digital literacy and digital access presented barriers, in some cases preventing ongoing participation in those who stated they deeply enjoyed the face-to-face sessions.

The consort diagram may have been different if the methods of delivery had been known from the start. Some people who declined participation due to difficulties physically accessing the hospital may have participated. However, those for whom digital delivery poses barriers may have declined. Clearly digital access is a vital consideration to address to overcome this potential barrier to participation. Additionally, participant rapport building appears to be an area requiring particular consideration.

Some limitations to this study are important to discuss. First, the need for rapid adaptation of study delivery meant that the methods for supporting singing online had not been refined. That said, it has provided a unique opportunity to gain insights into the transition from face-to-face to online delivery which has, by necessity, become widespread. Second, given the novelty of the online delivery, the session content and technical considerations are likely to develop over time with experience, which could alter the relevance of the current findings to future sessions. However, this is not necessarily a weakness, as the current findings provide useful results on which to base these developments. Third, the sample size was small due to the circumstances in which it was decided to evaluate this group and because the mode of delivery changed part-way through. This limits the confidence in quantitative impacts, and means it is unclear whether singers experienced benefits during the face-to-face or online part of the programme, or a combination of the two. Nevertheless, as a convenience pilot study, it provides useful indications of impacts, as well as informing future research. Similarly, the suboptimal attendance during online sessions limits the extent to which impact can be assessed, although when combined with the interview feedback this provides useful information regarding barriers and facilitators to participation that can be addressed in both practice and future research. See tables 2 and 3 for suggestions. Finally, it is important to consider the context in which this trial took place. The developing COVID-19 pandemic was a considerable source of concern for many people with COPD, who were identified as being at an increased risk of severe COVID-19 or death. Though all the participants lived in London, the situation and their response to it, is likely to have differed between individuals, which intern, may have shaped their experience of the intervention.

Despite the necessary, yet unusual, adaptations to the methods, this study has provided interesting and potentially useful results to inform the development of further research regarding online singing group delivery and research. These findings are useful for existing SLH groups who are moving to online delivery of previously in-person sessions. They also provide some of the first research findings to support the delivery of participatory online arts-in-health interventions in the context of COVID-19-related physical distancing.

The findings may also provide relevant insight for other related activities making an online transition such as pulmonary rehabilitation and Tai-Chi,^35^ and dance for people with long-term medical conditions. Many of these activities had begun to develop and test online delivery approaches prior to the COVID-19 pandemic,^36 37^ though the importance and potential utility of online delivery has now clearly increased.^17^

Further research should include larger studies assessing the health and well-being impact of online group singing in patient groups and for the wider population. Larger studies of SLH specifically, both online, and face-to-face (when appropriate to do so) remain a priority. Even after the acute phase of the COVID-19 pandemic online delivery of singing groups presents an opportunity to widen access to certain groups of people. In-depth qualitative research exploring participant experience would also be valuable, and in particular, in what ways the wider context of physical distancing measures impacts the experience of in person or online singing sessions.

## Conclusions

In conclusion, this study suggests that group singing sessions that have had to change delivery from face-to-face to online may produce clinically significant impacts on depression scores and improve balance confidence in people with COPD. The findings also identify important differences between online and face-to-face delivery including technological barriers for online delivery, and overcoming physical access barriers to face-to-face delivery. Despite a general preference for face-to-face sessions, online delivery was still felt to provide substantial health and well-being benefits. Future research on digitally delivered singing groups is required.
